# Evaluation of Non-Invasive Sampling Methods for Detection of Hepatitis E Virus Infected Pigs in Pens

**DOI:** 10.3390/microorganisms11020500

**Published:** 2023-02-16

**Authors:** Marina Meester, Aniek Rademaker, Martijn Bouwknegt, Renate W. Hakze-van der Honing, Arjan Stegeman, Wim H. M. van der Poel, Tijs J. Tobias

**Affiliations:** 1 Farm Animal Health Unit, Department of Population Health Sciences, Faculty of Veterinary Medicine, Utrecht University, 3584 CL Utrecht, The Netherlands; 2 Vion Food Group, 5281 RM Boxtel, The Netherlands; 3 Wageningen Bioveterinary Research, 8221 RA Lelystad, The Netherlands; 4 Royal GD, 7418 EZ Deventer, The Netherlands

**Keywords:** HEV, pig farms, sensitivity, specificity, boot sock, oral fluid, pooled faecal droppings, refinement, animal experiments, three Rs

## Abstract

Pigs are a reservoir of hepatitis E virus (HEV), which causes hepatitis in humans. To study the epidemiology of HEV in pig farms, sampling methods are currently used that cause discomfort to pigs, such as rectal sampling. In line with the 3Rs principle, we aimed to evaluate non-invasive methods to detect pens with HEV-shedding pigs. Twenty-eight pens of one farm were sampled cross-sectionally. Individual rectal swabs (IRS) were collected to determine prevalence within pens. Four pen-level samples were compared: a pool of IRS per pen (P), boot socks (BS), oral fluid (OF) and pooled faecal droppings (FD). Each sample was tested by RT-PCR and the sensitivity and specificity of each method was determined by Bayesian latent class analysis. According to IRS, 19/28 pens were HEV positive. BS had a sensitivity of 95% and detected HEV in pens with 10% of pigs shedding; however, specificity was below 30%. FD were comparably accurate to P, with a sensitivity and specificity of 94% and 86%, respectively. BS sampling is thus advised to detect early shedding of HEV or pen contamination, and FD to determine the duration of shedding. This study demonstrates that non-invasive sampling can replace rectal swabs in research on HEV in pigs.

## 1. Introduction

Hepatitis E virus (HEV) genotype 3 is an emerging zoonosis in industrialized countries [1], with more than 20,000 confirmed human cases in Europe between 2005 and 2015 and a sharp increase in cases between 2011 and 2015 [2]. Humans can become infected by direct or indirect contact with pigs and through the consumption of raw or undercooked pork liver [3]. Domestic pigs are a major reservoir of HEV, as HEV can be found on most pig farms worldwide [4]. Many aspects of the epidemiology of HEV in primary pig production have yet to be uncovered, for instance the source of infection of a herd, the routes of transmission between groups of pigs within farms, and the effectiveness of interventions applied to prevent infection of pigs [5]. Consequently, to better understand the infection dynamics of HEV in primary pig production, more research is needed.

Currently, research on HEV in primary pig production is primarily performed using invasive sampling methods to collect blood and faeces, via venipuncture and rectal swabs, respectively (e.g., [6,7,8,9,10]). Both methods require the handling and restraining of pigs, accompanied by stress and potentially pain [11,12]. In accordance with the 3Rs ethical principle (replacement, reduction, and refinement) [13], and in line with Directive 2010/63/EU of the European Parliament on protection of animals used for scientific purposes [14], accurate non-invasive sampling methods to study HEV in pig farms should become available.

Non-invasive sampling methods to investigate swine viruses are upcoming, not only because of welfare reasons, but also because they are inexpensive, simple, and safe. Examples include the collection of oral fluids via cotton swabs or chewing ropes, nasal wipes, processing fluids extracted from tails or testicles of newborn pigs, and faeces from boot socks or fresh droppings [15]. Pigs shed HEV in faeces and possibly urine [16] and HEV has been found in salivary glands [17], so oral fluids, boot socks and faecal droppings seem the most promising methods to replace invasive sampling. Oral fluid samples can be used to detect HEV RNA or antibodies [18,19,20]. Boot socks are mainly used to detect *Salmonella* spp., in both poultry and pig farming [21,22] and have also been shown to be useful for HEV RNA detection [23]. For determination of the HEV status on the farm level, faecal dropping samples for HEV RNA detection have already proved to be accurate compared to serum sampling [24]. Yet, for the determination of HEV shedding on the pen-level, no comparison of accuracy between sampling methods has been reported.

Our hypothesis is that non-invasive sampling methods comprise a tool at least as accurate as rectal-swab sampling, to determine HEV shedding in pens of pigs. Therefore, the aim of this study was to determine the sensitivity (Se) and specificity (Sp) of four different sample types tested with the same reverse transcription PCR (RT-PCR) test to classify a pen as positive when it contains at least one HEV-shedding pig. The sample types included pooled individual rectal swabs (P of IRS) (invasive), and pooled faecal droppings (FD), boot socks (BS) and oral fluid (OF) (non-invasive).

## 2. Materials and Methods

### 2.1. Farm Selection and Description

A cross-sectional study was conducted on a conventional fattening pig farm in December 2021. This pig farm was selected for having a high probability of HEV detection due to an estimated HEV seroprevalence of 77% at slaughter according to a study conducted in 2019 [25]. The farm houses about 5600 fattening pigs that originate from a farrow-to-grower farm owned by the same farmer, but located elsewhere. Pigs are transported to the fattening farm at around twelve weeks of age.

**Figure 1:**
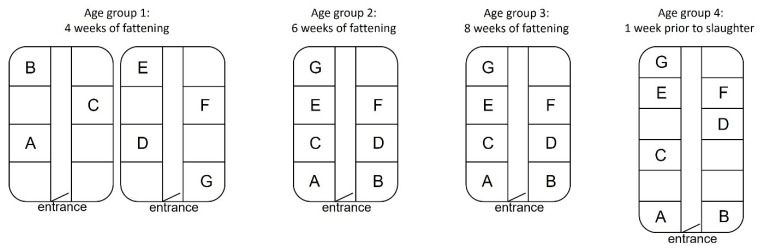
Location of sampled pens within farm compartments, per age group.

Four age groups were selected based on an expected different pen-level prevalence of HEV: a low prevalence at four weeks after the start of the fattening phase (group 1), intermediate to high at six and eight weeks after the start of the fattening phase (groups 2 and 3, resp.), and almost or completely negative one week prior to slaughter (group 4). The different prevalences were needed to compare IRS results to pen-level results and to have multiple populations in the Bayesian model (read below). The expectations were based on previous pilots on five farms, where individual and pen-level prevalence were determined in pigs of six age groups. The youngest selected age group was housed in two farm compartments, whereas the other age groups were housed in one compartment (Figure 1). Per age group, seven pens (A–G) were included, as according to previous experience individual rectal sampling of 25 to 30 pens with four people is manageable in one day. Pens of different locations across the compartment were selected, if possible (Figure 1).

The compartments of group 1 to 3 were in the same barn and all pens within these compartments were of equal size. The pens in group 1 contained between 25 and 27 pigs and groups 2 and 3 contained between 14 and 17 pigs. The compartment of group 4 was in a separate barn and contained more, yet smaller pens, with 10 to 12 pigs per pen.

### 2.2. Sampling Protocol

Pens were sampled by two teams of two persons to consecutively collect FD, BS, and IRS. To prevent contamination of samples and transmission of HEV between pens, disposable gloves and boot covers were changed per pen, and hairnets were changed per farm compartment.

Per pen, five swabs of fresh faeces of approximately 1 g were taken from individual droppings on the floor and collected in a 50 mL tube (Sarstedt, Nümbrecht, Germany) tube to a pooled FD sample [26]. The choice of five droppings per pen was arbitrary.

BS samples were collected by treading through the faeces-covered floor and purposefully stepping into accumulated faecal material [21]. Afterwards, socks were collected individually in a plastic zipper bag [26].

Furthermore, IRS were collected from all pigs per pen up to a maximum of 20. Sterile cotton swabs were inserted intrarectally for at least five seconds and the procedure was repeated if no faecal material was visible on the swab. A swab was indicated as contaminated when another pig or the pen environment was touched, and was then substituted by a new swab.

The collection of OF occurred asynchronous from the faecal sampling methods to prevent distraction of pigs while chewing on the ropes. The approach was similar to that described by Schott et al. (2021). Per pen, one 3-strand twisted, 12-mm diameter, unbleached cotton rope was fastened to the pen fence for 30 min, 10 cm above shoulder height of the pigs, away from drinking water and feed. The chewing ropes were fastened simultaneously for each pen per compartment. After removing the rope, it was squeezed and at least 3 mL of OF was collected in a 5 mL tube [20]. All samples were packed in a polystyrene box containing freezer packs and transported to the laboratory for direct processing.

### 2.3. Laboratory Protocol

IRS were transferred to a 15 mL tube and suspended in 2 mL tryptose phosphate 2.95% with gentamycin. FD samples were suspended in the same medium to obtain a 10% solution, followed by vortexing. After incubation for 30 min to soften the faeces in both the IRS as the FD samples, the tubes were vortexed for the second time, and centrifuged at 2500× *g* for 10 min. One mL of supernatant was transferred to a micronic tube and stored at −80 °C [26]. Per pen, 40 µL of supernatant from the IRS were combined to pooled IRS samples (P).

The BS samples were weighed, and the weights corrected for the known standard weight of dry socks by subtraction. The socks were suspended in PBS to obtain a 20% solution and processed in a Stomacher for one minute. One mL of supernatant was transferred to a micronic tube and stored at −80 °C [26]. OF samples were centrifuged for 10 min at 2000× *g* and the supernatant was stored at −80 °C [18]. One week later, all samples were transported on dry ice for an hour to another laboratory, frozen at −80 °C again, and used for RNA extraction and detection that same week.

RNA extractions from all samples were done with the Quick-DNA/RNA Viral magbead Kit (Zymo Research, Irvine, CA, USA), according to the manufacturer’s instructions. Briefly, 600 µL viral DNA/RNA buffer with 20 µL magnetic beats was added to each 300 µL sample containing 1:1 RNA/DNA shield. The RNA isolation was performed using the PurePrep 96 (Molgen) automated DNA/RNA purification system and the RNA was eluted in 50 µL DNAse/RNAse-free water. On each plate of the RNA isolation, a positive and negative process control was added.

HEV RNA was detected by real-time RT-PCR assays, using Taqman FastVirus 1-step master mix as described in Jothikumar et al. (2006) [27]. For TaqMan^®^ RT-PCR, the 20 µL reaction contained 5 µL of RNA, 5 µL of 4× Taqman FastVirus 1-step master mix (appliedbiosystems)), 8.8 µL H_2_O, 0.5 µL of primer F (10 µM), 0.5 µL of primer R (10 µM, and 0.2 µL of probe P (10 µM) at concentrations of 250, 250 and 100 nM, respectively. The LightCycler480 (Roche, Basel, Switzerland) was used for all real-time RT-PCR tests. Reverse transcription was carried out at 50 °C for 5 min, followed by denaturation at 95 °C (20 s). RNA was amplified immediately with 45 PCR cycles at 95 °C (10 s), 60 °C (30 s). PBS was included as negative control and HEV genotype 3 from hepatocytes of an infected pig as positive PCR control, and the controls were included in each run. A Cp-value < 40 was deemed positive for HEV RNA.

### 2.4. Bayesian Latent Class Analysis

The Se and Sp of the pooled samples were estimated by a latent class analysis (LCA) in a Bayesian framework. LCA is used in case of absence of a gold standard. Although the IRS can be viewed upon as a gold standard, one cannot be sure of the correct classification, because HEV can be absent by chance in the equivalent volume of faeces tested if the pig is shedding at low levels. RNA-extraction and PCR methods used to determine the status of the individual swabs are not perfect, HEV shedding may be intermittent, so with a single swab from an infected pig, one may miss the true HEV infection status, and not all pigs per pen can be rectally swabbed in the case of large groups of pigs. In LCA, one assumes that membership in an unobserved or ‘latent class’, can be explained by patterns across multiple ‘tests’. Thereby, true prevalence of one or more populations, along with the performance of the tests can be estimated. In the current study, the ‘latent class’ contained at least one HEV-shedding pig (in the pen) and the ‘test’ was the combination of a sampling method with the RT-PCR [28]. The Bayesian framework enables the addition of prior information from previous research about for instance the prevalence in a population, and the use of Markov Chain Monte Carlo (MCMC) simulation, to estimate the joint posterior distribution of prevalence and test method performance (Se and Sp).

To make a Bayesian LCA model identifiable, the degrees of freedom must be equal to or exceed the number of parameters to be estimated [29]. To that end, the pens were divided into populations based on the four age groups. This yielded an identifiable model with 12 parameters to be estimated and 28 degrees of freedom. Aside from a model with conditional independence between the four sampling methods, a model with conditional covariance between FD and P results was performed, as with both methods, faecal swabs are taken from multiple pigs per pen (giving two extra parameters to be estimated).

The models were run in OpenBUGS v. 3.2.3, that uses Gibbs sampling as an MCMC method. Models were performed with minimally informative beta-distribution priors for the Se and Sp (Table 1, model 1) and with both minimally and weakly informative priors for the prevalence per age group, based on previous studies in the literature [6,30] and the HEV status of pigs from this farm at slaughter in a previous study [6,25] (Table 1, model 2), to assess the effect of adding prior beliefs to the posterior distributions. No strong priors for Se and Sp were included, as hardly any information in the literature about the performance of the sampling methods is available, and the model is identifiable without strong priors. Three independent chains were run for 10,000 iterations, with a burn-in period of 1000 iterations. Convergence was evaluated by visual examinations of trace plots of the three chains, and by checking the Brook-Gelman-Rubin (bgr) diagnostic [31,32]. The evaluation of adding conditional dependence between FD and P results was based on the 95% posterior credible intervals (PCI) of covariance of the Se and Sp. Conditional dependence was taken out of the model in case the 95% PCI contained the value 0.

## 3. Results

### 3.1. Results of Individual Rectal Samples

Nineteen pens (68%) were HEV positive according to IRS, all in age groups 1, 2 and 3, i.e., four, six and eight weeks after the start of the fattening phase. Pigs that would be slaughtered within one week post sampling, group 4, were all HEV negative according to IRS. In age group 2, all 112 sampled pigs were shedding HEV, and the prevalence of excreting pigs in group 3 was, on average, 97%. In group 1, within-pen prevalence ranged from 0% to 60% in positive pens (Table S1).

### 3.2. Results of Pen-Level Sampling Methods

**Figure 2:**
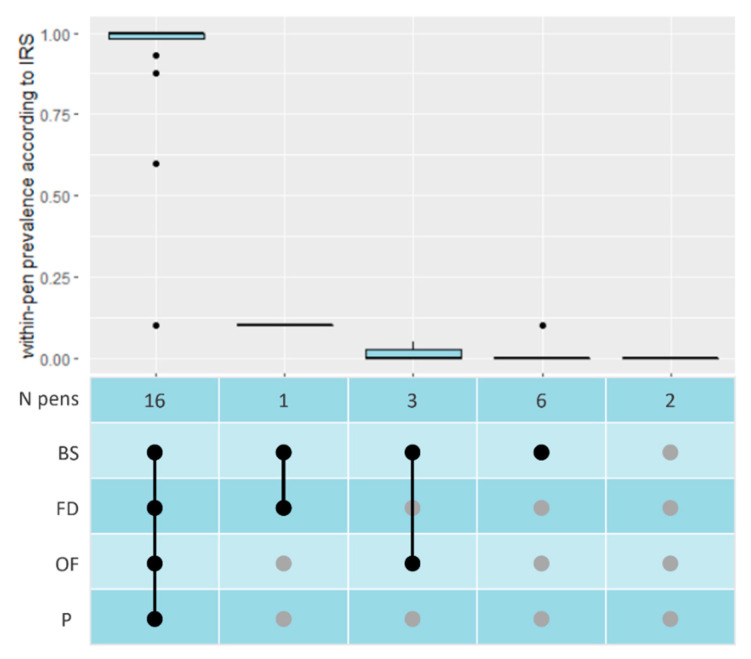
Within-pen prevalence according to IRS, for all combinations of positive tests for the four sample types. (N = number of, IRS = Individual Rectal Swabs, BS = Boot Sock, FD = Faecal Dropping, OF = Oral Fluid, P = Pooled Individual Rectal Swabs, **●** = positive PCR test, **●** = negative PCR test).

Sixteen pens tested PCR positive according to all four sampling methods and two were negative according to all methods (Figure 2). For age groups 2 and 3, full consensus was found between results of the pen-level sampling methods, with all pens having tested HEV positive in each sample type (Table S1). Cp-values between 21.4 and 33.4 were found. The highest Cp-values, i.e., the smallest number of HEV RNA copies, belonging to the two pens with less than 100% of pigs shedding HEV. Generally, the lowest Cp-values were found with BS samples. In age groups 1 and 4, only four out of fourteen pens had consensus, i.e., the same sample types tested positive for these pens. BS were positive most often (Figure 2).

### 3.3. Bayesian Latent Class Analysis

Convergence could be assumed, and the burn-in period of 1000 iterations was considered adequate according to visual inspection of the trace plots and the bgr diagnostic. Adding conditional covariance between FD and P samples retrieved 95% PCI that included a value of 0, so the final model does not comprise dependencies between sampling methods. The use of more informative priors for the prevalence per age group did slightly alter the posterior prevalence estimates; however, the estimates of the Se and Sp of all sampling methods were not affected. Therefore, the results of the model with minimally informative priors and with conditional independence between all sampling methods are presented here (Table 2). BS samples had the highest Se of 95%, and the lowest Sp of 22%. FD and P had similar sensitivities, 94%, but the Sp of P was slightly higher than FD, according to the LCA.

## 4. Discussion

The aim of the study was to determine the Se and Sp of non-invasive pen-level sampling methods compared to a pool of rectal swabs, to assess whether a non-invasive method can replace rectal-swab sampling for HEV detection in pens with pigs.

We have found that a pool of five fresh FD tested with RT-PCR to detect pens with at least one HEV-shedding pig gave an estimated Se and Sp above 85%. We therewith found the FD method comparable to the results of pooled IRS collected within pens. Collecting five droppings yields a probability of about 40% of having at least one positive dropping in the pool at a within-pen prevalence of 10%, increasing to nearly 100% for a within-pen prevalence above 60%. This was indeed reflected in our results, with negative results for the droppings only when the within-pen prevalence based on IRS was lower than or equal to 10%. Correlating the number of droppings to the number of pigs per pen may further increase the Se.

BS and OF samples had an Se of 95 and 94%, although Sp was lower than that of the pooled rectal swabs, being around 20 and 70%, respectively. This means that the probability of false-positive results for HEV shedding in pens was increased. Possibly, BS can take up faeces from previously shedding pigs. This could be excretion by the pigs currently present in the pen or by pigs from a previous round, when cleaning and disinfection was inadequate and HEV was sufficiently persistent to remain detectable. It has been demonstrated before that HEV can be detected in recently cleaned and disinfected pig pens [33]. In that way, BS are not only related to HEV shedding, but also to environmental contamination, and could be useful to identify HEV-positive farm compartments or farms. For OF, the exact mechanisms and dynamics of HEV excretion are undetermined, but HEV RNA has been detected in salivary glands and tonsils [17,34,35,36] and the excretion in OF was described previously [18]. Additionally, noting the faecal-oral transmission route, finding HEV in the oral cavity was not unexpected. The positive OF results for two pens with pigs almost entering slaughterhouse while being negative in IRS, may indeed be the result of oral uptake of faeces containing HEV RNA during rooting behavior of the pigs. On the other hand, the individual rectal swabs may also have been negative for HEV because pigs may intermittently shed HEV in faeces, or because insufficient faecal material was collected with the swabs. In that case, Sp of BS and OF would be higher than those estimated by the Bayesian LCA. An indication for this is that the Cp-values of BS were a few points lower than of P, suggesting that in case of a low number of HEV particles, P could miss HEV shedding.

The assumption in Bayesian LCA that testsensitivity and -specificity are equal across populations may have been violated, because the populations in the current study were different age groups. Pigs with a higher age may have been infected for a longer duration of time, which can lead to different levels of shedding compared to younger pigs [37]. The magnitude of this effect depends on the dose-dependent Se of the diagnostic method to detect a single RNA particle and the dose-dependent probability of RNA particles ending up in the tested sample. Given the generally high probability of a positive test result with a few RNA particles in RT-PCR and the order-of-magnitude-excretion of virus particles throughout infection, we do not expect this effect to alter our conclusions. Furthermore, the age gap is relatively small, so the assumption seems justified. The assumption of different prevalences across populations was, in hindsight, correct based on IRS, and the assumption of conditional independence was assessed in the model.

Although only one farm was included in the study, we deem extrapolation of the results to other farms, breeds, or age groups, possible. The sampling methods can be applied to each pig farm, regardless of such farm factors. Preferably, pens with an intermediate prevalence would also have been included. Inclusion of those pens may have slightly altered the results, for instance because the BS samples may have distinguished a lower proportion of pens false-positively. However, the high and low within-pen prevalences found in this study strengthen the distinctiveness between the sampling methods and have served the purpose of determining which sample can replace invasive sampling.

All in all, FD samples were nearly equal to rectal-swab sampling in the detection of HEV-shedding pigs within pens, although pens with a few HEV-shedding pigs may be missed. The probability of the latter can be reduced by increasing the numbers of droppings per pen to more than five. BS have a low Sp and pick up HEV long after infection seems to have passed, but will pick up HEV infection in pens with less than 10% of shedders. Accordingly, we recommend BS sampling for early detection of HEV-shedding pigs, and for investigating environmental contamination. In conclusion, non-invasive sampling by FD and BS are accurate to replace rectal-swab sampling and comprise cost- and labor-effective, but most of all animal-friendly, methods for HEV research in pig farms.

## Figures and Tables

**Table 1:** Priors used in Bayesian LCA (latent class analysis), with α and β as parameters for the beta distribution (Se = sensitivity, Sp = specificity, Prev. = prevalence, SD = standard deviation).

	Model 1 Minimally Informative Priors				Model 2 Weakly Informative Priors			
Model Parameter	α	β	Mode	SD	α	β	Mode	SD
Se (of 4 methods)	1	1	0.5	0.29	1	1	0.5	0.29
Sp (of 4 methods)	1	1	0.5	0.29	1	1	0.5	0.29
Prev. age group 1	1	1	0.5	0.29	6	14	0.3	0.1
Prev. age group 2	1	1	0.5	0.29	18	2	0.9	0.07
Prev. age group 3	1	1	0.5	0.29	12	8	0.6	0.1
Prev. age group 4	1	1	0.5	0.29	2	18	0.1	0.07

**Table 2:** Posterior estimation of sensitivity, specificity of four pen-level sampling methods and of prevalence per age group, according to a Bayesian LCA with minimally informative priors and conditional independence between sampling methods (PCI = posterior credibility interval, BS = boot sock, FD = faecal dropping, P = pool of individual rectal swabs, OF = oral fluid).

	Parameter	Median (%)	95% PCI
Pen-level Prevalence	Age group 1	33.7	8.24–66.1
	Age group 2	88.8	62.6–99.7
	Age group 3	88.8	63.0–99.7
	Age group 4	11.3	0.31–37.1
Sensitivity	BS	94.5	80.8–99.8
	FD	94.4	80.3–99.8
	P	94.1	79.3–99.8
	OF	94.3	80.0–99.8
Specificity	BS	21.7	5.13–46.2
	FD	85.9	63.9–98.4
	P	92.7	74.9–99.8
	OF	71.2	45.4–90.9

## Data Availability

This study was conducted in accordance with the EU regulations on animal experimentations (2010/63/EU) and approved by the Dutch Central Committee of Animal Experimentation (CCD) under license number AVD1080020197664.

## Supplementary Materials

The following supporting information can be downloaded at: https://www.mdpi.com/article/10.3390/microorganisms11020500/s1, Table S1: Results of IRS and pooled sampling methods per pen, including both pen status and Cp-value per pooled sample type. (IRS = Individual Rectal Sampling, BS = Boot Sock, FD = Faecal Dropping, P = Pooled Individual Rectal Swabs, OF = Oral Fluid, Undet. = undetected/ Cp-value > 40).
