# Supplementary material for: Evaluation of Non-Invasive Sampling Methods for Detection of Hepatitis E Virus Infected Pigs in Pens
Source: Microorganisms. 2023 Feb 16;11(2):500. doi: 10.3390/microorganisms11020500 (PMC9962119; doi:10.3390/microorganisms11020500)
Supplement: Supplementary file 1 [file microorganisms-11-00500-s001.zip › microorganisms-2217809-supplementary.pdf]

microorganisms

Supplementary Material

Table S1. Results of IRS and pooled sampling methods per pen, including both pen status and Cp-value per pooled sample type. (IRS = Individual Rectal Sampling, BS = Boot Sock, FD = Faecal Dropping, P = Pooled Individual Rectal Swabs, OF = Oral Fluid, Undet. = undetected/ Cp-value >40).

Age category	Pen	Prevalence HEV IRS	Number of pigs sampled	BS	FD	P	OF	BS Cp-value	FD Cp-value	P Cp-value	OF Cp-value
1	A	0.6	20	1	1	1	1	31.78	36.92	33.93	35.16
1	B	0.05	20	1	0	0	1	39.32	Undet.	Undet.	39.67
1	C	0	20	1	0	0	0	36.12	Undet.	Undet.	Undet.
1	D	0.1	20	1	1	0	0	36.61	36.83	Undet.	Undet.
1	E	0.1	20	1	0	0	0	35.30	Undet.	Undet.	Undet.
1	F	0	20	0	0	0	0	Undet.	Undet.	Undet.	Undet.
1	G	0.1	20	1	1	1	1	32.85	32.98	37.19	36.36
2	A	1	16	1	1	1	1	24.52	27.49	26.97	24.01
2	B	1	15	1	1	1	1	23.67	23.38	27.03	26.75
2	C	1	17	1	1	1	1	22.89	25.23	28.30	27.00
2	D	1	16	1	1	1	1	22.94	22.93	27.15	26.04
2	E	1	16	1	1	1	1	22.80	22.42	26.38	24.65
2	F	1	16	1	1	1	1	22.83	23.58	27.23	26.45
2	G	1	16	1	1	1	1	23.01	24.97	27.08	26.25
3	A	1	15	1	1	1	1	21.76	23.83	27.19	26.11
3	B	0.875	16	1	1	1	1	29.09	28.92	33.28	31.25
3	C	1	15	1	1	1	1	23.51	24.82	28.39	26.92
3	D	1	14	1	1	1	1	22.68	23.54	28.80	27.28
3	E	1	16	1	1	1	1	21.43	26.70	27.63	26.35
3	F	0.933	15	1	1	1	1	26.64	28.47	33.06	29.32
3	G	1	15	1	1	1	1	23.28	25.70	26.82	27.51
4	A	0	12	1	0	0	0	37.13	Undet.	Undet.	Undet.
4	B	0	11	1	0	0	0	38.40	Undet.	Undet.	Undet.
4	C	0	12	1	0	0	1	35.15	Undet.	Undet.	36.79
4	D	0	12	1	0	0	0	38.94	Undet.	Undet.	Undet.
4	E	0	11	1	0	0	0	34.62	Undet.	Undet.	Undet.
4	F	0	12	0	0	0	0	Undet.	Undet.	Undet.	Undet.
4	G	0	11	1	0	0	1	38.85	Undet.	Undet.	39.50
